# Sexual Functioning and Opioid Maintenance Treatment in Women. Results From a Large Multicentre Study

**DOI:** 10.3389/fnbeh.2019.00097

**Published:** 2019-05-14

**Authors:** Lorenzo Zamboni, Anna Franceschini, Igor Portoghese, Laura Morbioli, Fabio Lugoboni, GICS Group, Arzillo C.

**Affiliations:** ^1^Department of Medicine, Verona University Hospital, Verona, Italy; ^2^Addictive Behaviors Department, Local Health Authority, Treviso, Italy; ^3^Department of Medical Sciences and Public Health, University of Cagliari, Cagliari, Italy; ^4^Gruppo InterSerT di Collaborazione Scientifica (GICS), Verona, Italy

**Keywords:** methadone, addiction, quality of life, women sexuality, buprenorphine

## Abstract

Opioid maintenance treatment (OMT) is the most widespread therapy for both females and males opioid addicts. While many studies have evaluated the OMT impact on men’s sexuality, the data collected about the change in women’s sexual functioning is still limited despite the fact that it is now well-known that opioids - both endogenous and exogenous - affect the endocrine system and play an important role in sexual functioning. The present study aims to determine how OMT with buprenorphine (BUP) or methadone (MTD) affects sexual health in women; examining also any possible emerging correlation between sexual dysfunction (SD), type of opioid and patients’ mental health. This multi-center study case recruited 258 female volunteers attending Italian public Addiction Outpatients Centers that were stabilized with OMT for at least 3 months. SD was assessed with the Arizona Sexual Experience Scale. The twelve-item General Health Questionnaire was used to assess participants’ mental health conditions. The results show that 56.6% of women receiving OMT for at least 3 months presented SD without significant differences between MTD e BUP groups. The majority of the subjects with SD have a poorer quality of intimate relationships and worse mental health than the average. To the best of our knowledge, the present study is the largest report on the presence of SDs in women as a side effects of MTD and BUP used in OMT. Since SDs cause difficulties in intimate relationships, lower patients’ quality of life and interfere with OMT beneficial outcomes, we recommend that women undertaking an opioid therapy have routine screening for SD and we highlight the importance to better examine opioid-endocrine interactions in future studies in order to provide alternative potential treatments such as the choice of opioid, opioid dose reduction and hormone supplementation.

## Introduction

The World Health Organization (WHO) describes sexual health as a state of physical, emotional, mental and social well-being in relation to sexuality itself and recognizes it as a right to every human being (World Health Organization [WHO], 2006). It is commonly accepted that a healthy sexuality is fundamental to one’s sense of self-worth (Kaplan, 1974); it represents the integration of the biological, emotional, social, and spiritual aspects of who one is and how one relates to others (Covington, 1997).

Despite its proven capital importance for human self-esteem, sexuality has been a neglected topic in scientific research and in treatment of women with Substance Use Disorders (SUD) to this day. Reproduction intended as a combination of contraception, pregnancy, parenthood and risky sexual behavior (i.e., sexually transmitted diseases and prostitution), is the aspect drawing most of the attention in terms of research and special health services for women. Although these aspects deserve priority in regards to feminine sexuality, they are not fully exhaustive on the matter.

The lack of professional study cases on the relationship between SUD and sexual functioning appears more evident considering sexual health in opioid dependent women under Opioid Maintenance Treatment (OMT) on buprenorphine (BUP) and methadone (MTD). To this day we have a serious lack of data in regards to this topic despite the fact that opioids -both endogenous and exogenous- evidently affect the endocrine system (Katz and Mazer, 2009; Rhodin et al., 2010; Voung et al., 2010) and play an important role in sexual functioning (Palha and Esteves, 2008).

Sexual dysfunctions (SDs) are a frequent adverse effect during opioid treatment for both men and women however, most of the studies had been conducted basing the researches on male candidates receiving OMT (Brown and Zueldorff, 2007; Lugoboni et al., 2017).

To date most of the studies that have analyzed the impact of opioid treatment on female sexuality had been conducted on women treated with opioid analgesics aimed to cure non-malignant chronic pain. These studies demonstrated that opioids inhibit the production of multiple hypothalamic, pituitary, ovarian and adrenal hormones, causing opioid-induced hypogonadotropic hypogonadism that can determine amenorrhea or hypomenorrhea, SDs, fatigue and depression in female patients (Daniell, 2008; Rhodin et al., 2010).

Opioid maintenance treatment combined with psychosocial interventions is the most widespread treatment for opioid dependence. In Europe MTD is currently the most prescribed medicine against heroine dependency: 69% of opioid dependent patients are undergoing this treatment, meanwhile the 28% of the subjects is assuming BUP. Among 700.000 European patients in OMT, 20% is represented by women (EMCDDA, 2015).

Given the fact that one of the main goals of the OMT is the amelioration of the patients’ quality of life and their reintegration in a gratifying social life, it is clear how assessing and curing eventual SDs in both female and male OMT patients is of primary importance. It is also demonstrated that iatrogenic sexual disorders can act against treatment retention and achievement of a good quality of life for the patients (Xia et al., 2013).

Taking into account the limited number of monitored and rigorous studies regarding OMT and SDs as a side effect of this therapy in women, the present study puts its focus on the presence of SDs in Italian female patients treated for opioid dependence with MTD or BUP in specialized outpatient centers. The authors hypothesize that OMT impacts the sexual health of opioid addicted women, as it is already ascertained in men, and they aim to examine if there is any correlation between possible SD, type of opioid, daily dose administration and patients’ mental health. Due to the factors explained above, the results of this study could help to find evidence-based models that would allow assisting clinicians to address and treat sexual issues and related concerns with aimed therapy.

## Materials and Methods

The study was conducted in 20 Addiction Treatment for Outpatients Centers of the Italian public health system. The philosophy of intervention, policies and procedures applied were similar in each Center and the accessibility threshold was the same across all structures.

Italian Addiction Treatment Services provide outpatient treatment programs with a variety of therapeutic and rehabilitative strategies: MTD, BUP, and naltrexone are administered in association with possible psychosocial interventions, such as psychotherapy, family therapy, group therapy, social support and medications for psychiatric co-morbidity.

The selected centers did not differ in the psychosocial treatment protocols associated with MTD and BUP, or in the admission criteria. In the Italian Addiction Services the majority of patients are heroin addicts. There are no exclusion criteria regarding the access to the public health system. Patients who fail to respond to interventions such as OMT and continue to inject heroin are not dismissed by these centers.

Between 1st July and 31st December, 2015, a cross-sectional survey was administered to a large sample of patients receiving MTD or BUP maintenance treatment for heroin dependence. The sample included 258 women between the age of 18 and 61 (mean age: 37) enrolled in a drug recovery program in treatment centers for clinically diagnosed heroin dependence (American Psychiatric Association, 2000) DSM IV TR. Patients were receiving either MTD (N 198, 76.7%; mean daily dose 60.5 mg) or BUP (N 56, 23.3%; mean daily dose 10.8 mg) maintenance in combination with psychosocial treatment. At the time of the study they had been stabilized with an OMT for at least 3 months.

Underage patients, subjects following a drug-free treatment or an opioid substitution therapy for less than 3 months (and/or other than MTD/BUP) were excluded from the study case and also those who presented difficulty of language comprehension.

The questionnaires and data sheets were delivered by a nurse to the participating patients. This was done to optimize patients’ privacy and to minimally affect responses, as nurses are less involved in therapy compared to doctors and psychologists. Patients filled the questionnaires at the facility or at home and handed the documents anonymously. As indicated by a previous focus group among surveyed patients the collection of the questionnaires was carried out using an urn and not by direct delivery to the staff. Patients gave a written informed consent in order to take part to this survey, which was approved by the Public Health System ethical committee of Verona University Hospital in Verona, Italy. All participants were volunteers and were not paid for their participation. The patients could stop the survey’s compilation at any time. Study procedures did not interfere with the daily protocols of the centers.

### Measures

Sexual dysfunction was assessed by the Arizona Sexual Experience Scale (ASEX) (McGahuey et al., 2000). It is composed by five items rated on a 6-point Likert-type scale, with higher scores reflecting greater or lower dysfunction level. Each item quantifies a major domain of sexual function, sexual drive, psychological arousal, physiologic arousal (vaginal lubrication for women), ability to reach orgasm, and orgasm satisfaction (e.g., “How easily are you sexually aroused?”). Cases of SD were established according to three criteria: (a) a total score ≥19; (b) any item with an individual score ≥5; and (c) any three or more items scoring ≥4 were considered as SD (McGahuey et al., 2000). These three criteria showed optimal sensitivity and specificity for SD. The main dependent variable for all the analyses will use cases applying all those criteria (0, “not SD,” 1 “SD”), and analyses will be repeated for each specific criterion in order to test possible differences with more restrictive definitions. In the present study, the internal reliability was 0.86.

The twelve-item General Health Questionnaire (GHQ-12) was used to assess the mental state of the participants. This tool is intended to screen for general (non-psychotic) psychiatric morbidity (Goldberg and Williams, 1988; e.g., “Have you recently felt you couldn’t overcome your difficulties?”). It has been widely used and translated into many languages and extensively validated in general and clinical populations worldwide (Werneke et al., 2000). Items were answered on a 4-point scale from 0 (not at all) to 3 (much more than usual).

Higher scores indicate poorer mental health. In the present study, the internal reliability was 0.89.

The questionnaire also included demographic and drug related variables such as age, marital status, education level, and use and dosage of MTD and BUP. The respondents completed anonymously all questionnaires.

### Statistical Analysis

At first, differences in proportions or means between patients with or without SD were compared with chi-square tests (categorical variables) and *t*-tests for unrelated samples (continuous).

In order to assess the strength of the associations with categorical variables the Cramer’s V coefficient of association (range from 0 for no association to 1 as perfect association) was calculated, whereas the effect size (Hedge’s g) was measured on associations with continuous variables.

Hedge’s g provides values that are very similar to Cohen’s d [d = g/sqrt (N/df)] for which the following arbitrary rules of thumb are often used: 0.2–0.3, small effect; 0.5, moderate effect; and 0.8, large effect (Cohen, 1988).

A confirmatory factor analyses (CFA) was carried out for examining the distinctiveness of the scales used in this study. More specifically, we compared a full measurement model to a one-factor structure (where items were set to load into a common factor). The model ft was tested considering the Comparative Fit Index (CFI), the Incremental Fit Index (IFI), and the Root-Mean-Square Error of Approximation (RMSEA). According to Kline (2005) and Byrne (2016), the CFI and IFI values should have a cutoff value of ≥0.90, and RMSEA a value of ≤0.08 to indicate a good ft of the model. Reliability analysis was performed using Cronbach’s α measure.

Finally, to examine whether demographics (age, marital status, and education level), use of MTD and BUP, and overall psychological well-being were predictive of sex dysfunction, a stepwise multiple regression analysis was carried out. A *P*-value <0.05 was considered statistically significant.

Statistical analyses were carried out using PASW Statistics 18⋅0 and AMOS 16⋅0 (Chicago, IL, United States, Arbuckle, 2007).

## Results

### Factorial Validity of the Scales

Results from CFA showed that the hypothesized two-factor model (χ2 = 297.25 *df* = 113, *P* < 0.01, RMSEA = 0.079, CFI = 0.92, IFI = 0.92) fits the data significantly better than the one general factor model (χ2 = 606.26, *df* = 114, *P* < 0.01, RMSEA = 0.130, CFI = 0.78, IFI = 0.78) providing evidence of discriminable different factors.

### Descriptive Statistics

A total of 56.6% (*SE* = 3.1; *n* = 146) of the sampled patients manifested SD considering the three criteria explained above. 18.6% (*SE* = 2.4; *n* = 48) fulfilled criterion a, 43.0% criterion b (*SE* = 3.1; *n* = 111), and 43.4% criterion c (*SE* = 3.1; *n* = 112). In the total sample the mean age was 37.7 years (*SD* = 10.6; range: 18–61); 61.3% of the subjects were single, 15.2% married, 18.4% divorced or separated and 5.1% widowed; 69.0% presented a secondary education level while the 26.4% had a higher education, and 4.7% an elementary level of education. 76.4% (*n* = 197) were taking MTD with an average of 60.6 mg (*SD* = 73.5) and 20.6% (*n* = 53) were taking BUP with an average of 10.5 mg (*SD* = 7.6). The mean score in the GHQ for the total sample was 14.6 (*SD* = 7.0).

Comparisons between those with and without SDs indicated that women with SDs were older, were more often separated or divorced, had lower levels of education, assumed higher doses of MTD (among those consuming the drug), and presented a poorer mental health as measured by the GHQ12. No differences among groups were found in regards to the percentage of patients taking MTD or BUP or the doses of this last drug. All these results are summarized in Tables 1, 2.

**Table 1:** Characteristics of the sample according to the presence of sexual dysfunction.

Variables	Sexual dysfunction *N* = 146	Not sexual dysfunction *N* = 112	*p*	*g (95% CI) or V*
Age	39.1 (11.1)	35.5 (9.9)	**0.006**	**0.34 (0.09,0.59)**
*Marital status (%)*				
Married	13.2 (2.8)^∗^	17.9 (3.6)^∗^	**0.048**	**0.176**
Widowed	6.9 (2.1)^∗^	2.7 (1.5)^∗^		
Divorced/separated	22.9 (3.5)^∗^	12.5 (3.1)^∗^		
Single	56.9 (4.1)^∗^	67.0 (4.5)^∗^		
*Education level (%)*			**0.004**	**0.209**
Elementary	6.9 (2.1)^∗^	1.8 (1.3)^∗^		
Secondary	74.0 (3.6)^∗^	62.5 (4.6)^∗^		
Higher	19.2 (3.3)^∗^	35.7 (4.5)^∗^		
*Methadone (% yes)*	80.1 (3.3)^∗^	71.4 (4.3)^∗^	0.103	0.102
*Methadone, mg*	69.4 (91.1)	47.6 (30.3)	**0.041**	**0.30 (0.01,0.58)**
*Buprenorphine (% yes)*	17.2 (3.1)^∗^	25.0 (4.1)^∗^	0.127	0.095
*Buprenorphine, mg*	8.9 (8.2)^∗^	12.0 (6.7)^∗^	0.132	0.41 (-0.13,0.95)
*GHQ*	16.0 (7.1)	12.8 (6.4)	**<0.001**	**0.47 (0.22,0.42)**


*Values between brackets are standard deviations unless indicated: ^∗^, Standard errors. Comparisons: t-tests for continuous variables, chi-square tests for categorical variables g, Hedge’s effect size, V, Cramer’s V. Values in bold are statistically significant (p < 0.05).*

**Table 2:** Results of logistic regression analyses, persons without sexual dysfunction (reference category) vs. persons with sexual dysfunction, unadjusted and controlling for age (except for the age effect).

Variables	OR, unadjusted (95% CI)	OR, adjusted (95% CI)
Age	1.03 (1.01,1.06)	
*Marital status (ref.: single)*		
Married	0.87 (0.43,1.75)	0.73 (0.35,1.51)
Widowed	3.05 (0.81,11.50)	2.15 (0.54,8.53)
Divorced/separated	**2.16 (1.07,4.34)**	1.56 (0.72,3.36)
*Education level (ref.: elementary)*		
Secondary	0.31 (0.07,1.45)	0.33 (0.07,1.58)
Higher	**0.14 (0.03,0.69)**	**0.16 (0.03, 0.80)**
*Methadone (ref.: No)*	1.61 (0.91,2.87)	1.70 (0.95,3.07)
*Methadone, mg*	**1.01 (1.001,1.02)**	**1.01 (1.0001,1.02)**
*Buprenorphine (ref.: No)*	0.62 (0.34,1.15)	0.58 (0.31,1.07)
*Buprenorphine, mg*	0.94 (0.87,1.02)	0.94 (0.87,1.02)
*GHQ*	**1.07 (1.03,1.12)**	**1.09 (1.04,1.13)**


*Numbers in bold indicate statistically significant effects (p < 0.05).*

Interestingly, when performing the same analyses using more restrictive definitions of SD, results did not change for criteria b (any one item with a score ≥5) and c (any three or more items with scores ≥4), while in regards to criteria a (cut-off score in the total scale ≥19) there was no difference shown in the severity of mental health symptoms.

As shown in Table 3, only one significant effect emerged for item # 2 (easiness for sexual activation) when analyzing distribution of scores for specific ASEX items in women taking or not taking MTD. Subjects taking MTD reported higher difficulty for getting aroused, with an average effect size (Cramer’s *V* = 0.286). The total ASEX score, however, did not significantly differ [*t*(256) = 0.15; *p* = 0.882] between those taking MTD (mean = 14.77, *SD* = 4.58) or BUP (mean = 14.67, *SD* = 4.51).

**Table 3:** Association between responses to specific items from the ASEX and the use of MTD (% of persons using MTD responding to each category).

Items	1	2	3	4	5	6	*p*	*V*
How strong is your sex drive?	1.52	12.69	22.34	31.47	21.83	10.15	0.210	0.167
How easily are you sexually aroused (turned on)?	**1.53**	**10.71**	**20.92**	**39.80**	**21.94**	**5.10**	**0.001**	**0.286**
How easily does your vagina becomes moist or wet during sex?	6.12	22.45	25.51	28.57	12.76	4.59	0.265	0.159
How easily can you reach an orgasm?	1.80	15.32	27.03	33.33	16.22	6.31	0.146	0.146
Are your orgasms satisfying?	9.91	31.53	29.73	16.22	7.21	5.41	0.071	0.259


*From 1 “extremely strong/ easily/ satisfying” to 6 “no sex drive/ never/ can’t reach orgasm.” Higher scores means more sexual dysfunction. Numbers in bold indicate statistically significant effects (p < 0.05); tests: chi-square (df = 5).*

### Hierarchical Regression Analyses

Stepwise multiple regression analysis was conducted with individual characteristics as shown in Table 4, including age, marital status, and education level, use of MTD and BUP, and overall psychological well-being as predictor variables and sex dysfunction as criterion (dependent) variables. Table 4 shows that the model accounted for 6.5% of the criterion variance.

**Table 4:** Stepwise linear regression analysis of predictors of sexual dysfunction (*N* = 258).

Variables	B	SE	ß	*P*-value
Age	0,008	0,03	0,019	0,784
Marital status	0,33	0,271	0,085	0,224
Education level	0,109	0,3	0,023	0,716
MET	-1,566	1,605	-0,147	0,33
BUP	-1,743	1,684	-0,155	0,302
Overall psychological well-being	0,176	0,041	0,271	0,000


*Predictors of sexual dysfunction final model produced at p = 0.05, F = 3,85, P < 0.01, R^2^ = 0.063.*

Overall psychological well-being was the only significant predictor (β = 0.27, *p* < 0.001).

## Discussion

The scientific literature that treats gender differences in SUD is rather recent and it highlights substantial differences between men and women. It is demonstrated by these studies that gender influences the prevalence, the origin, the progression and the outcome of these disorders. Women showed a quicker transition from use to dependence (Becker and Hu, 2008), worse clinical conditions at the time of admission, more frequent comorbidity for depression and anxiety, increased suicide risk, and worse physical health compared to men presenting opioid use disorder. Psychiatric comorbidity often precedes and favor onset of SUD in women, such as post-traumatic stress disorder which is found related to physical and sexual abuse in all ages and worst socioeconomic conditions (Cotto et al., 2010; Eiroá-Orosa et al., 2010; Back et al., 2011). Women indulge in sexual risky behavior more than men by avoiding condom use, choosing a greater number of sexual partners and using sex in exchange of money and/or drugs more frequently; women also tend to choose stable partners with SUD (Quaglio et al., 2004, 2006). They often accept unprotected sex in order to grant the continuity of the relationship (Sheeran et al., 1999). Numerous studies verified that intimate partner violence and childhood sexual abuse in general population are strongly related to risky sexual behaviors and to the occurrence of sexually transmitted diseases (Urada et al., 2013). These dynamics facilitate the manifestation of unbalanced love or sexual relationships that favor the masculine partner’s power. Together with SUD these situations jeopardize women’s determination to look for and find a healthy sexual life (Engstrom et al., 2012; Gilbert et al., 2015).

To this day gender studies have largely neglected the sexual aspects of opioid dependent women and to the best of our knowledge, the present study is the largest report on women with SD on MTD or BUP maintenance treatment. It focuses on the sexual health of 258 women in OMT using consistent and validated measures of SD and also evaluating other factors (i.e., demographic data, mental health, and opioid dose), that could contribute to SD. The results show that 56.6% of women receiving BUP or MTD for at least 3 months show SD without significant differences between MTD e BUP groups.

These results differ from the ones reported by Moreira et al. (2008), in a large community survey that showed how 30.1% of adult women in Southern Europe (Italy, Spain, France) suffer from lack of sexual interest, while 22.7% experience lack of sexual pleasure and 24.8% incur inability to reach orgasm. These percentages indicate that female patients in OMT have a higher rate of SDs in comparison to the general population.

In the present study the MTD group shows a significantly higher excitation disturbance compared to the BUP group while considering specific ASEX issues. These results are consistent with those of the study conducted by Giacomuzzi et al. (2009), which demonstrated how, in a small sample of 30 women in OMT, it is harder to reach orgasms while taking MTD instead of BUP.

Furthermore, demographic variables emerged from this study, BUP and∖or MTD intake are not significant predictors of SDs, and the majority of subjects with SD have a quality of intimate relationship and mental health poorer than the average. The results from the stepwise regression show how women’s overall psychological well-being is positively linked to SD. These findings are consistent with those of other studies reporting SD, anxiety and depression in women treated with opioid in chronic pain (Daniell, 2008; Katz and Mazer, 2009). The relationship between mood disorders and SD is actually still unsettled in women in OMT, but in many cases it could be directly associated with opioid-induced hypogonadotropic hypogonadism, especially for impaired androgen production. The testosterone opioid-induced suppression can have important consequences other than SD, such as potential anxiety, depression, fatigue and a generally reduced quality of life. These symptoms were reported to have improved with androgen supplementation in women undergoing long-term opioid treatment (Brown and Zueldorff, 2007; Katz and Mazer, 2009). As a matter of fact, the presence of depression, anxiety and a generally reduced quality of life are common in women in OMT and could be due to associated conditions and co-morbidities (i.e., other medications, primary psychiatric disorders, other medical conditions, use of other substances low socioeconomic status), regardless of the opioid treatment. In case of co-presence of these symptoms and SDs, female patients in OMT should be assessed for opioid-induced hypogonadism by laboratory endocrine evaluation to investigate if alterated gonadal hormon levels play any role in SDs and in mood and/or anxiety disorders.

Furthermore, demographic variables taking BUP and∖or MTD were not significant predictors of SD.

It is important to mention the correlation between MTD dose and SD emerged by this study, dynamic which is not present in BUP groups. Other studies have shown a dose-response effect in patients undergoing MTD treatment due to boosting testosterone suppression by increasing the dose of MTD. This result is clearer in men than in women, due to limited scientific information on testosterone levels in female patient undergoing MTD treatment (Bawor et al., 2014). Our outcomes are in line with the previous study carried out by Parvaresh et al. (2015) that used ASEX and focused on MTD dose-related effect in sexual functioning in adult women. Conversely there is no evidence in literature of a link between SD and BUP dosage in women in OMT treatment or about testosterone level in these subjects. If further studies on women will confirm the correlation between SDs and MTD dosages and on the contrary no correlation with BUP dose, this issue should be taken into account at the moment of choice of opioid medication, especially because there are findings that women need higher MTD doses compared to man in order to avoid quitting the treatment (Vigna-Taglianti et al., 2016). The reason behind this last result is still unclear, hypothetically it could be partially associated to the evidence that higher MTD dosages are requested in patients diagnosed with post traumatic stress disorder or depression (Trafton et al., 2006). These illnesses are more frequent in women than in men as explained above. Moreover it should be noted that, despite the lack of evidence in literature of the correlation between the severity of SUD and MTD or BUP dosages needed in OMT, higher MTD doses are predictive of major reduction in illicit opioid consuming in both men and women (Fareed et al., 2009).

## Conclusion

Sexual dysfunctions may cause difficulties in intimate relationships, lower patients’ quality of life, can favor and maintain the SUD, interfere with OMT beneficial outcomes and influence adherence to treatment (Brown and Zueldorff, 2007; Xia et al., 2013; Bawor et al., 2014). It is important to explore the cause of SDs through a multidimensional evaluation. It is very important to inform patients on the possible side effects of opioid therapy on their sexuality and when present, to their treatment also. Spreading the information can avoid the arousal of negative thoughts about themselves and their sexual self-efficacy.

This study shows how OMT can determine sexual side effects in women despite being an essential and effective treatment in opioid addicted patients. Unfortunately, the lack of evidence about SD in women in OMT implicates absence of intervention models in case of sexual disturbances. This can be an obstacle to clinicians to carefully enquire about sexual health in these subjects. Women on opioid therapy should have routine screening for SD longitudinally, and should be treated with appropriate measures.

In the light of the above-mentioned considerations, we now understand the necessity of continuing the studies in order to overcome the existing limited literature about opioid induced SD in women and therefore better examine hypogonadism in women in OMT. The aim is to provide female patients the chance to eventually apply potential treatments like the choice of opioid, opioid dose reduction and androgen supplementation.

## Limitations of the Study

Whereas the strength of the present study is its larger sample size compared to previous researches, the limitations concern the questionnaire as it is self-reported and the definition of SD as it is subjective. A lack of sexual activity, for example, is not always perceived as SD; personal views (i.e., cultural, religious, or other) often bias interpretation. Furthermore this research lacks a longitudinal perspective. As this research was cross-sectional, we were unable to analyze causal influence and changes in the studied variables across time.

## Data Availability

All datasets generated for this study are included in the manuscript and/or the supplementary files.

## Author Contributions

FL was responsible for the study concept and design. GICS contributed to the data acquisition. LZ assisted with the data analysis and interpretation of findings. AF and LM drafted the manuscript. All authors critically reviewed the content and approved the final version of the manuscript for publication.

## Conflict of Interest Statement

The authors declare that the research was conducted in the absence of any commercial or financial relationships that could be construed as a potential conflict of interest.
